# GATA transcription factor in common bean: A comprehensive genome-wide functional characterization, identification, and abiotic stress response evaluation

**DOI:** 10.1007/s11103-024-01443-y

**Published:** 2024-04-17

**Authors:** Mohamed Farah Abdulla, Karam Mostafa, Abdullah Aydin, Musa Kavas, Emre Aksoy

**Affiliations:** 1 Faculty of Agriculture, Department of Agricultural Biotechnology, Ondokuz Mayis University, 55200 Samsun, Türkiye; 2 The Central Laboratory for Date Palm Research and Development, Agricultural Research Center (ARC), 12619 Giza, Egypt; 3 Faculty of Arts and Sciences, Department of Biology, Middle East Technical University, 06800 Ankara, Türkiye

**Keywords:** *GATA* transcription factor, Common bean, Abiotic stress response, Functional characterization, Gibberellic acid

## Abstract

The GATA transcription factors (TFs) have been extensively studied for its regulatory role in various biological processes in many plant species. The functional and molecular mechanism of GATA TFs in regulating tolerance to abiotic stress has not yet been studied in the common bean. This study analyzed the functional identity of the *GATA* gene family in the *P. vulgaris* genome under different abiotic and phytohormonal stress. The *GATA* gene family was systematically investigated in the *P. vulgaris* genome, and 31 PvGATA TFs were identified. The study found that 18 out of 31 *PvGATA* genes had undergone duplication events, emphasizing the role of gene duplication in GATA gene expansion. All the *PvGATA* genes were classified into four significant subfamilies, with 8, 3, 6, and 13 members in each subfamily (subfamilies I, II, III, and IV), respectively. All PvGATA protein sequences contained a single GATA domain, but subfamily II members had additional domains such as CCT and tify. A total of 799 promoter *cis*-regulatory elements (CREs) were predicted in the *PvGATAs*. Additionally, we used qRT-PCR to investigate the expression profiles of five *PvGATA* genes in the common bean roots under abiotic conditions. The results suggest that *PvGATA01/10/25/28* may play crucial roles in regulating plant resistance against salt and drought stress and may be involved in phytohormone-mediated stress signaling pathways. *PvGATA28* was selected for overexpression and cloned into *N. benthamiana* using Agrobacterium-mediated transformation. Transgenic lines were subjected to abiotic stress, and results showed a significant tolerance of transgenic lines to stress conditions compared to wild-type counterparts. The seed germination assay suggested an extended dormancy of transgenic lines compared to wild-type lines. This study provides a comprehensive analysis of the *PvGATA* gene family, which can serve as a foundation for future research on the function of GATA TFs in abiotic stress tolerance in common bean plants.

**Supplementary Information:**

The online version contains supplementary material available at 10.1007/s11103-024-01443-y.

## Key message

This study comprehensively explored the GATA transcription factor family in the common bean genome, identifying 31 PvGATA genes and uncovering their role in abiotic stress tolerance. Notably, PvGATA28 demonstrated enhanced stress resistance in transgenic lines, offering promising insights for further research in common bean plant resilience.

**Supplementary Information:**

The online version contains supplementary material available at 10.1007/s11103-024-01443-y.

## Introduction

*Phaseolus vulgaris* L., commonly known as common bean, navy, pinto, red kidney, or French beans, is a significant source of protein for global populations. The crop’s popularity stems from its noncentric, flavorful nature and adaptability (Gepts et al. 1986; Myers and Kmiecik 2017). Moreover, the common bean is a vital component of healthy diets owing to its exceptional nutritional content and functional properties (Yang et al. 2018). The common bean is cultivated under different climatic conditions and produced in developed and developing countries (Beaver and Osorno 2009). Recently, many researchers have conducted studies on common beans’ protein, fat, fatty acid, mineral content (Celmeli et al. 2018), polyphenols (Yang et al. 2018), vitamin and mineral content (AUGUSTIN, BECK, KALBFLEISH, KAGEL, & MATTHEWS, 1981), trypsin inhibitors activity (TIA), phytic acid, tannins, ascorbic acid, thiamine, and protein (Sangronis and Machado 2007) in terms of food security and nutrition. One of the most critical challenges facing the world in the next three decades is achieving food security. Moreover, climate change is likely to have an adverse impact on agricultural production and primary crop nutrition. Extensive crop modeling studies have demonstrated that common bean growing regions and yields will be negatively affected by 2050 (Hummel et al. 2018).

The availability of numerous plant genomes due to the rising number of next-generation sequencing (NGS) platforms has created an opportunity to comprehend the evolutionary history of plants and determine the role of transcription factors (TFs) in plant biology. Plant genomes contain various transcription factor families, for ecample, *WRKY* (Jiang et al. 2017), *MYB* (Ambawat et al. 2013; Kavas et al. 2022), *DREB* (Khan 2011), *bZIP* (Alves et al. 2013), *MADS*-box (de Folter et al. 2005; Kavas et al. 2022), *bHLH* (Kavas et al. 2016), and *GATA* (Kim et al. 2021; Liang et al. 2023; Reyes et al. 2004; Wang et al. 2023). GATA TFs, are found widely among eukaryotes like mammals, fungi, and plants (Dobrzycki et al. 2020; Lowry and Atchley 2000; Romano and Miccio 2020). They play vital roles in plant development and response to environmental stresses. They are characterized by a highly DNA-binding domain that recognizes a consensus motif WGATAR (W = T or A; R = G or A) in the promoter regions of their target genes (Lowry and Atchley 2000; Reyes et al. 2004). Recent studies demonstrated their crucial regulatory roles in diverse biological processes, including seed germination, embryogenesis, chloroplast development, flowering time, carbon and nitrogen assimilation, and responses to various biotic and abiotic stresses (An et al. 2019; Jiang et al. 2020, 2021; Liu et al. 2019; Schwechheimer et al. 2022). *GATA* genes actively particibate in plant development and respond to environmental challenges by regulating downstream genes through DNA binding (Jiang et al. 2021).

Furthermore, The GATA TFs have a significant function in diverse abiotic stresses in plants. Numerous *GATA* factors were found to be upregulated or downregulated in soybean leaf in response to low nitrogen stress. Two *GATA* factors, *GmGATA44* and *GmGATA58*, were shown to be involved in nitrogen metabolism regulation in soybean plants (Zhang et al. 2015). Under salinity conditions, *OsGATA8* regulates the expression of critical genes involved in stress tolerance, scavenging reactive oxygen species, and chlorophyll biosynthesis (Nutan et al. 2020). Under different nitrogen levels, the poplar GATA TF *PdGNC* can control chloroplast ultrastructure, photosynthesis, and vegetative development in Arabidopsis (An et al. 2014). Therefore, GATA TFs are important regulators of plant growth and development, as well as adaptation to different environmental conditions.

Little information on the *GATA* genes and their role under abiotic stress in *Phaseolus vulgaris* is available. Herein, we performed genome-wide identification, phylogenetic studies, expression level analysis, and identification of amino acid patterns and transmembrane helix of 31 *GATA* genes in common bean. Additionally, the function of the *PvGATA28* gene was evaluated in transgenic tobacco plants grown under abiotic stress conditions. This study serves as a valuable resource for gaining a deeper comprehension of the structure and functionality of *PvGATA* genes.

## Materials and methods

### Retrieval and characterization of *GATA* genes in *P. vulgaris*

A comprehensive and non-redundant data set of common bean proteins containing the conserved GATA domain was compiled using BLAST and keyword searches. Firstly, The Hidden Markov Model (HMM) and BLASTP algorithms were used to identify PvGATA proteins (Finn et al. 2015). Additionally, we conducted a keyword search in the Phytozome v13 database (https://phytozome-next.jgi.doe.gov/) to identify additional possible *GATA* genes that were possibly missed in the first step. The candidate sequences of GATAs were confirmed using InterPro (https://www.ebi.ac.uk/interpro/) and Pfam (http://pfam-legacy.xfam.org/) tools (Hunter et al. 2008; Mistry et al. 2020). The query proteins and nucleotide sequences of all putative *PvGATA* genes were obtained from (https://phytozome-next.jgi.doe.gov/). Putative *PvGATA* genes were named based on their arrangement order on chromosomes of the *P.vulgaris* genome. Moreover, the length of amino acids, molecular weights (MW), and isoelectric point (pI) of GATA proteins were calculated using ExPASy (http://www.expasy.ch/tools/pi_tool.html). The PROSOII tool was used to predict the solubility of candidate GATA proteins based on their sequences (Jiang et al. 2021).

### Multiple sequence alignment and phylogenetic analysis

The protein sequence of each putative *GATA* genes from *Phaseolus vulgaris*, *Arabidopsis thaliana, and Oryza sativa* were acquired from the Phytozome v13 database for the phylogenetic structural analysis and synteny analysis for the determination of inter and intra-species relationship. A phylogenetic tree derived from multiple sequence alignment was carried out by the ClustalW 2.0 program in MEGAX software. The neighbor-joining method-based phylogenetic tree was constructed using the bootstrap test (1000 replicates) and the Jones-Taylor-Thornton (JTT) model in the IQtree web tool. Phylogenetic trees were visualized with ITOL v3 (http://itol.embl.de/).

### Chromosomal localization, motifs, CREs, and gene structures in common bean

Using a generic feature format version 3 (GFF3) obtained from the Phytozome v13 database, the chromosomal position of *PvGATA* genes was examined. The TBTools software was employed to locate *PvGATA* genes on their corresponding chromosome locations. We examined the gene structure (exons and introns) as well as conserved domains and motifs to demonstrate the potential link between the evolution process of *PvGATA* genes and their structure-function correlation. TBtools (Chen et al. 2011) was utilized to visualize the motifs of *Phaseolus vulgaris* GATA proteins predicted and analyzed by MEME. The selected parameters specified that each sequence could contain zero or one contributing motif site, with a total of six repeated motifs chosen. The widths of the motifs were set to a range of 6 to 50, while the remaining parameters were kept at their default values (Wu et al. 2016). To ensure accuracy, each motif was individually evaluated, and only those with an e-value of less than 1e-10 were considered for motif detection in GATA proteins *of Phaseolus vulgaris*. To analyze the *cis*-acting regulatory elements (CRE), the upstream 2000 bp DNA sequences from the translation initiation site of each *PvGATA* gene were extracted from the Phytozome v13 database and submitted to the PlantCare web tool (http://bioinformatics.psb.ugent.be/plantcare/html). The results were then visualized using the Tbtools software.

### Analysis of the evolutionary divergence of *PvGATA* genes family using gene duplication, synteny analysis, and protein-protein interaction

In this study, we utilized the One Step MCScanX method to perform multiple alignments using the genome sequence file and the genome structure annotation file of *P. vulgaris* downloaded from the Phytozome v13 database. We used the dual synteny plotter for the MCScanX program to visualize the collinearity results. These steps were conducted using the Tbtools software (Chen et al. 2020). To predict gene duplications of the *PvGATAs*, we employed the Plant Duplicate Gene Database (PlantDGD) and identified tandem repeats among duplicate genes located on the same chromosome (Qiao et al. 2019). Additionally, we conducted a BLASTP search to detect segmental duplications of the PvGATA proteins in the common bean and used the MCScan tool to determine their collinear blocks. To estimate the evolution of the *PvGATA* genes, we calculated the ratio between the nonsynonymous mutation rate and synonymous mutation rate (Ka/Ks) via TBtools software based on previous reports (Kavas et al. 2022). In short, we first blasted PvGATA proteins against the Phytozome v13 database and filtered the hits over 60% sequence similarity threshold to estimate the evolutionary relationship of these proteins. We then created a tab-delimited text file to calculate Ka/Ks in TBtools software. To analyze the protein-protein interaction (PPI), we utilized the STRING web portal (http://string-db.org), and orthologs of these proteins were found in *Arabidopsis thaliana* (Szklarczyk et al. 2018). Finally, we investigated the PPI interaction of these proteins based on default settings.

### Plant material and qRT-PCR transcript expression analysis

This study used a locally cultivated commercial common bean variety (*Ispir*) for its known resistance to saline environments. *Ispir* seeds were surface sterilized in a 5% sodium hypochlorite solution before being planted in vermiculite-filled pots. Plants were cultivated in a fully controlled growth chamber at 24 C with a photoperiod of 16 h light and 8 h dark. After four weeks, the plants were treated to drought, salinity, and phytohormones (Indole acetic acid and Abscisic acid) treatments using polyethylene glycol 6000 (PEG), NaCl, ABA, and IAA, respectively. The plants were subjected to salt and drought stress by adding 200 mM NaCl and PEG (20%) to the Hoagland solution. Phytohormone treatment was done by spraying 100 µM ABA and 100 µM IAA on the leaves. Samples of stress and hormone-treated roots were taken at 6, 24, and 48 h following stress treatment and preserved at -80 C until use in RNA isolation. We extracted total RNA using the RNeasy Plant Mini Kit (Qiagen) according to the manufacturer’s instructions. The purity and concentration of the RNA were verified using a NanoDrop TM 2000/2000c spectrophotometer and a 1.5% (w/v) agarose gel. The iScript™ cDNA Synthesis Kit (Bio-rad, USA) was used to create the first strand cDNA. Phytohormone and stress-related expression of five randomly selected candidate *PvGATA* genes were measured with qRT-PCR analysis performed on the Agilent Mx3000P device with Solis BioDyne 5 × HOT FIREPol® EvaGreen® qPCR Mix Plus (ROX). In this study, *PvActin11* was employed as the reference gene for normalization in common bean, while *NbEFα1* served as the reference gene for transgenic *N. benthamiana* overexpressing PvGATA28, ensuring reliable and accurate expression (Kavas et al. 2020). All primers used for the qRT-PCR expression analysis are listed in Table S1. qRT-PCR conditions were carried out at 95 °C for 2 min, at 95 °C for 15 s, and 60 °C for 1 min. The 2^−∆∆CT^ technique was used to compute the relative expression.

### RNA-Seq-based in silico gene expression analysis of *P. vulgaris*

In this study, the expression levels of PvGATA TFs were investigated using RNA-seq from different publicly available datasets, which had been collected from various common bean genotypes under various tissues, growth stages, and abiotic and biotic stress. Raw data were obtained from the Sequence Read Archive (SRA) in the National Center for Biotechnology Information (NCBI) database, and transcriptome analyses were conducted using cloud bioinformatic tools such as CyVerse and Galaxy. HISAT2 was used to map the reads to the reference genome, while Stringtie 1.3.3 and Ballgown were utilized for transcript assembly and differential expression analysis, respectively. DEGs were identified as genes with a fold change value log2 > 1 and a *p*-value < 0.05. A heatmap was generated using TBTools to visualize the log2 fold change values. For stress expression analysis, seven different comparisons were conducted to determine the expression levels of all the *PvGATA* genes in diverse tissues and under various stress conditions. Firstly, two comparisons were made using RNA-seq data (PRJNA656794) from leaf and root explants of resistant (Ispir) and sensitive (T43) genotypes treated with salt stress. The third comparison was conducted using data from salt-stressed lower hypocotyl at the sprout stage of the Ispir genotype (PRJNA691982). The fourth comparison was made using RNA-seq data from drought-tolerant genotype Perola under drought stress versus control (PRJNA508605), while the fifth comparison was conducted using RNA-seq read values obtained from *P. vulgaris* plants infected with *Sclerotinia sclerotiorum* (PRJNA574280). Finally, the seventh comparison was made using the data collected from common bean plants under cold stress treatment (PRJNA793687). The tissue-specific expression patterns of *PvGATAs* were obtained from the publicly available transcriptome data (PRJNA210619).

### Construction of plant transformation vector

For generating the *PvGATA28* overexpression tobacco lines, the *PvGATA28* coding sequence (921 bp) was PCR-amplified with the forward primer 5’-CACCATGATACCAACTTATCG-3’ and reverse primer 5’-CTAAGTGATATAAAGATCTGAGG-3’. The *PvGATA28* was cloned into the plant expression vector using the pENTR Directional TOPO Cloning Kit from Invitrogen (UK). Initially, following the purification of the amplified *PvGATA28* PCR product, it was incorporated into the pENTR/D-TOPO vector as per the instructions provided by the manufacturer. The targeted *PvGATA28* sequence was then integrated into a destination vector (pIPKb004 Accession No. EU161570) through the Gateway® LR recombination reaction (Invitrogen UK). Positive colonies underwent screening using gene-specific primers and restriction digests. Final verification was accomplished by Sanger sequencing to confirm the insert and its orientation. The recombinant plasmid was then introduced into *A. tumefaciens* strain GV3010 using the MicroPulser electroporation system (Bio-Rad, USA). Tobacco leaves underwent a transformation process using the Agrobacterium-mediated transformation as described in (Kavas et al. 2020). After selection and molecular verification, we obtained two independent *PvGATA28* overexpressing lines.

### Analysis of subcellular localization of PvGATA28 by transient expression under confocal microscope

Transient expression was done on *Nicotiana benthamiana* plants grown in growth chamber set at 23–25 °C. The PvGATA28 coding sequence was attached to GFP’s C-terminal end in the pGWB6 vector using LR reaction (Nakagawa et al. 2007). The pGWB6 vector harboring GFP was used as negative control. This was followed by transformation in *Agrobacterium tumefaciens* (strain GV3101). A single colony culture was grown into a stable phase in Luria–Bertani medium enriched with selective antibiotics (Kanamycin and hygromycin) until the OD600 reached 0.6, then centrifugated at 5000 g for 5 min to pellet the cells. These were subsequently resuspended in MMA solution (comprising 10 mM MES at pH 5.6, 10 mM MgCl2, and 200 mM Acetosyringone) to achieve an OD600 of 1 and allowed to incubate at room temperature for 2 h. Leaves of *N. benthamiana* plants were then agroinfiltrated using a needleless syringe at 100 μm per spot. Following a co-cultivation period of 3–4 days in the plant growth chamber set at 23–25 °C, the agroinfiltrated plants were subjected to analysis for fluorescence (Hernández-Sánchez et al. 2015). We employed an excitation wavelength of 288 nm, with spectral detection parameters adjusted to range from 497 to 537 nm, to facilitate precise observation of sub-cellular localization through a Zeiss LSM700 confocal microscope (Carl Zeiss, Jena, Germany).

### Unveiling physiological dynamics in *N. benthamiana* lines overexpressing *PvGATA28*

Physiological assessments were performed to evaluate *PvGATA28* gene expression under salt and drought stresses. In short, T1 seeds of the *PvGATA28* overexpression line were sown in MS media supplemented with 150 mM NaCl and 150 mM mannitol for 8 weeks. Subsequently, the collected leaf materials were used for RNA extraction. Each experiment was replicated at least three times independently. Tests also included seed germination, MDA, and proline assays. *PvGATA28* transgenic lines and wild-type Tobacco seeds were grown on MS media supplemented with mannitol 150 mM and NaCl 150 mM (Secgin et al. 2022). Germination rates of seeds were recorded daily. Plant-free proline content was quantified using established protocols (Bates et al. 1973; Kavas et al. 2020). Malondialdehyde (MDA) levels were measured to gauge membrane damage from salt stress, following the thiobarbituric acid (TBA) method as outlined by (Hodges et al. 1999; Kavas et al. 2020). Leaf samples were used for these analyses. All experiments were repeated three times independently, and the average data was calculated.

### Statistical analysis

The data, represented as mean ± standard error from three independent biological replicates. *P*-Value was calculated using the One-way ANOVA in SPSS 26.0 (SPSS Inc., Chicago, IL, USA). Statistically significant differences are indicated as follows: *, *p* < 0.05 and **p < 0.01.

## Results

### Identification and characterization of *PvGATA* genes

We have successfully identified and isolated 31 *GATA* genes through a rigorous BLAST-P search conducted on both the common bean genome database in Phytozome V13 and the NCBI repository. It is important to note that we meticulously eliminated genes lacking the characteristic GATA domain. A comprehensive roster of these identified genes can be found in Table 1. In accordance with a systematic nomenclature protocol, we have designated these GATA TFs as *PvGATA*, bestowing them numerical designations corresponding to their positional arrangement on the chromosomes. The genes responsible for encoding these 31 PvGATA TFs exhibited varying amino acid lengths, spanning from 107aa (PvGATA3) to 544aa (PvGATA11), culminating in an average size of approximately 290 amino acids.

**Table 1:** Characterization of GATA proteins

Transcript ID	NCBI Accession	Gene Name	Chr No.	Strand	CDS (bp)	Protein Length (AA)	Protein M. Weight (kDa)	pI	GRAVY	Intron:Exons
Phvul.001G035600	XP_007161012.1	*PvGATA1*	1	reverse	873	291	32.01	9.61	-0.798	2:03
Phvul.001G109500	XP_007161927.1	*PvGATA2*	1	reverse	816	272	29.92	7.04	-0.596	1:02
Phvul.001G226500	XP_007163338.1	*PvGATA3*	1	forward	321	107	11.77	9.5	-0.489	1:02
Phvul.002G001500	XP_007156588.1	*PvGATA4*	2	forward	1617	539	59.29	5.97	-0.575	7:08
Phvul.002G030800	XP_007156947.1	*PvGATA5*	2	reverse	726	242	26.62	8.5	-0.819	1:02
Phvul.002G112000	XP_007157952.1	*PvGATA6*	2	reverse	960	320	35.2	5.9	-0.664	2:02
Phvul.002G213800	XP_007159158.1	*PvGATA7*	2	forward	1083	361	39.71	5.52	-0.799	11:12
Phvul.002G250800	XP_007159598.1	*PvGATA8*	2	forward	426	142	15.62	9.35	-0.544	2:03
Phvul.003G110400	XP_007154340.1	*PvGATA9*	3	reverse	489	163	17.93	9.45	-0.659	2:03
Phvul.003G137100	XP_007154661.1	*PvGATA10*	3	forward	930	310	34.1	9.48	-0.845	2:03
Phvul.003G224500	XP_007155707.1	*PvGATA11*	3	forward	1632	544	59.84	6.45	-0.552	7:08
Phvul.003G253900	XP_007156047.1	*PvGATA12*	3	forward	720	240	26.4	6.49	-0.885	1:02
Phvul.003G258900	XP_007156103.1	*PvGATA13*	3	forward	882	294	32.34	7.62	-0.477	2:02
Phvul.004G079200	XP_007151835.1	*PvGATA14*	4	reverse	762	254	27.94	8.94	-0.614	1:02
Phvul.004G083100	XP_007151876.1	*PvGATA15*	4	reverse	1011	337	37.07	5.66	-0.48	2:02
Phvul.005G134400	XP_007150193.1	*PvGATA16*	5	forward	972	324	35.64	6.71	-0.617	2:03
Phvul.007G095300	XP_007143712.1	*PvGATA17*	7	reverse	1065	355	39.05	6.11	-0.376	2:03
Phvul.007G227300	XP_007145298.1	*PvGATA18*	7	forward	753	251	27.61	6.95	-0.888	3:02
Phvul.008G157600	XP_007140987.1	*PvGATA19*	8	forward	1047	349	38.39	6.42	-0.724	1:02
Phvul.008G182900	XP_007141281.1	*PvGATA20*	8	forward	936	312	34.32	5.94	-0.695	7:07
Phvul.008G254600	XP_007142122.1	*PvGATA21*	8	reverse	903	301	33.11	6.46	-0.796	7:07
Phvul.009G003800	XP_007135927.1	*PvGATA22*	9	reverse	903	301	33.11	6.54	-0.589	2:02
Phvul.009G035300	XP_007136307.1	*PvGATA23*	9	reverse	1017	339	37.29	4.96	-0.75	9:10
Phvul.009G035400	XP_007136308.1	*PvGATA24*	9	reverse	831	277	30.47	5.85	-0.676	6:07
Phvul.009G077500	XP_007136825.1	*PvGATA25*	9	reverse	678	226	24.86	9.39	-0.648	1:02
Phvul.009G110400	XP_007137230.1	*PvGATA26*	9	reverse	960	320	35.2	5.84	-0.75	1:02
Phvul.009G118600	XP_007137337.1	*PvGATA27*	9	reverse	348	116	12.76	9.95	-0.75	4:04
Phvul.009G232700	XP_007138732.1	*PvGATA28*	9	forward	921	307	33.77	9.44	-0.846	2:03
Phvul.010G146300	XP_007135644.1	*PvGATA29*	10	forward	1044	348	38.28	6.21	-0.627	4:02
Phvul.011G039600	XP_007131760.1	*PvGATA30*	11	reverse	912	304	33.44	5.37	-0.58	1:02
Phvul.011G080200	XP_007132266.1	*PvGATA31*	11	reverse	888	296	32.56	9.23	-0.556	2:03

NCBI, National Center for Biotechnology Information; Chr, Chromosome; CDS, coding sequence; bp, base pair; A.A, Amino acid; kDa, kilo Dalton; pI, isoelectric point; GRAVY, grand average of hydropath

Furthermore, an examination of the fundamental physiochemical properties of PvGATA proteins revealed an isoelectric point (pI) spectrum ranging from 9.95 (PvGATA27) to 4.96 (PvGATA23), with an average pI of approximately 7.28. Additionally, the average molecular weight approximated 31.94 kDa, with the highest observed molecular weight of 59.84 kDa for PvGATA11 and the lowest of 11.77 kDa for PvGATA3. Notably, an analysis of the Grand Average of Hydropathy (GRAVY) for PvGATA proteins indicated negative values within the range of -0.9 to -0.38, signifying a characteristic non-polar and hydrophilic nature. Finally, our predictive assessments suggest that the subcellular localization of PvGATA proteins primarily resides within the nucleus, with the exception of PvGATA3, which is anticipated to be located extracellularly.

### Phylogenetic classification of *PvGATA*, their motif, gene structure, and conserved domain analysis

**Fig. 1:**
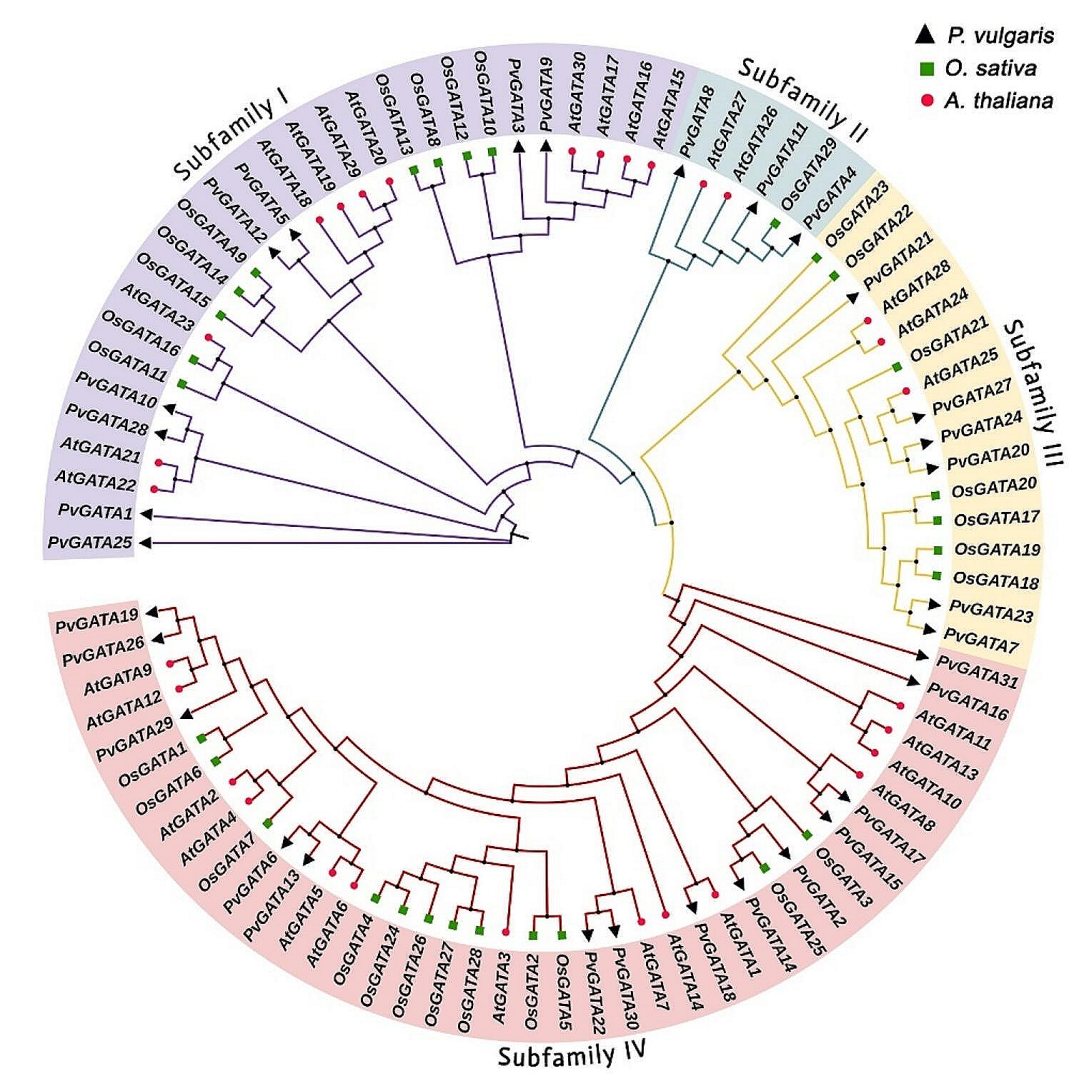
Phylogenetic relationship analysis of the GATA proteins between *Phaseolus vulgaris*, *Oryza sativa*, and *Arabidopsis thaliana*. Whole protein sequences of the GATA gene family were used for alignment using the MEGA X software. The phylogenetic tree was constructed using IQ-TREE 2 web tool using maximum likelihood with 1000 bootstrap replicates. Different colored branches correspond to distinct GATA subfamilies, and the GATA IDs of *Arabidopsis thaliana* and *Oryza sativa* were assigned based on previous studies

A maximum likelihood analysis was conducted to examine the inter- and intraspecific phylogenetic relationships among GATA TFs protein sequences in the common bean, *Arabidopsis*, and rice genomes. Following previous studies, the 31 *PvGATA* genes were grouped based on their conserved domains and motif structures (Fig. 1). As anticipated, the *PvGATA* genes were divided into four sub-families, namely subfamily I-IV. The analysis revealed that 8 *PvGATA* genes were in subfamily I, three genes in subfamily II, 6 in subfamily III, and 14 in subfamily IV.

**Fig. 2:**
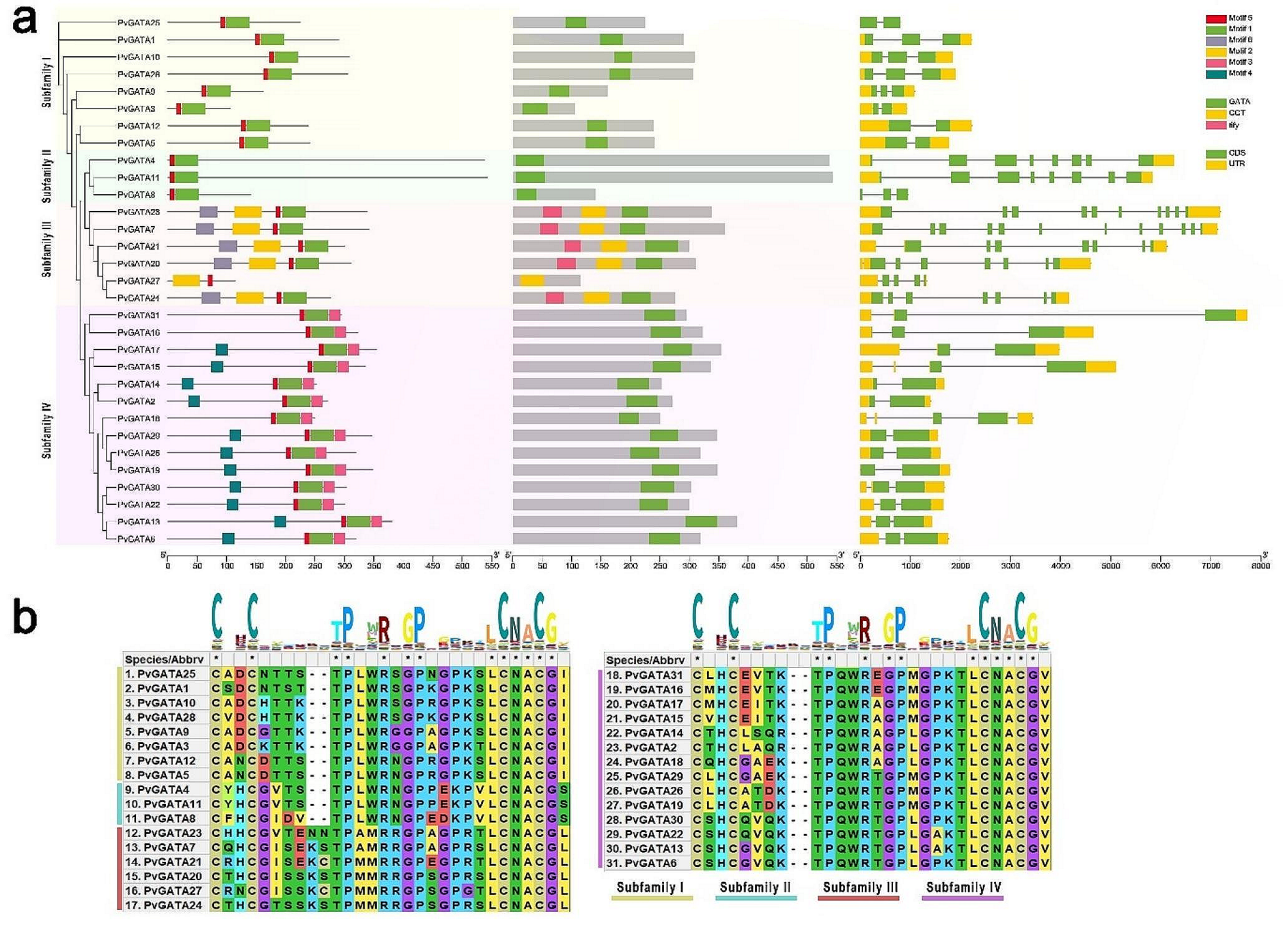
(**a**) The evolutionary relationships, conserved motifs, domain arrangement, and gene structures of the *PvGATA* TFs. A maximum likelihood phylogenetic tree was generated based on the full-length sequences of the *PvGATA* and 1000 bootstrap replicates using the IQTree webtool. The distribution of conserved motifs in *PvGATA* was predicted and was limited to 6 conserved motifs. Three conserved domains were found by analyzing the conserved domain structure of *PvGATA* sequences using the NCBI CD database. Moreover, the gene structures of *PvGATA* were analyzed and visualized, including the introns (black lines), exons (CDS, green rectangles), and untranslated regions (UTRs, yellow rectangles). The scale bar represented 100 bp. (**b**) The distribution and conserved regions of the GATA domain were investigated across all *PvGATA* proteins with respect to subfamilies using MEGA X software, with the HHM logo of the GATA domain shown

To compare the PvGATA TFs at both the nucleic acid and protein levels, we conducted a comprehensive analysis of the conserved motifs, gene structure, domains, and phylogenetic relationships among the 31 PvGATA proteins (Fig. 2a). Using the MEME search tool, we identified six conserved motifs labeled as motifs 1–5. The results showed varying compositions and distributions of these motifs among the PvGATA proteins, with the exception of PvGATA17, which had no assigned motifs. In contrast, motifs 1 and 5 were found in all the PvGATA proteins (excluding PvGATA17) and in the same arrangement. We also used the NCBI CD blast tool to analyze the conserved domains of the PvGATA proteins and found that the GATA domain was present in all 31 proteins. The *TIFY* domain and *CCT* (*CO, COL*, and *TOC1*) domains were present in subfamily II, while the *ASXH* domain was present in subfamily IV. Our comparison of the genetic exon and intron architecture of the *GATA* genes revealed that they had a varying number of exons, ranging from 2 to 13. Notably, subfamilies III and IV had a higher number of introns compared to the rest of the subgroups, which had 1–3 introns. Our analysis confirmed that there were similarities in motif structure, conserved domains, and exon/intron configuration among members of the same subfamily, further supporting our phylogenetic analysis and subfamily classification.

Furthermore, the GATA domain analysis yielded similar results to those found in *Arabidopsis* and rice (Reyes et al. 2004). All the subfamilies I-IV of PvGATAs exhibited 13 conserved residues in the zinc finger loop (C-X2-C-X18-C-X2-C). All 8 PvGATAs in subfamily I contained 21 residues along the residues in the zinc finger (C-X2-C-X20-C-X2-C) (Fig. 2b). Subfamily II had 22 conserved residues, subfamily III had 21 conserved residues, and subfamily IV contained 22 conserved residues. Moreover, several amino acid sites within the GATA domains, such as LCNACG residues, demonstrated high levels of conservation among common bean plant, *Arabidopsis*, and rice.

### Chromosomal localization, synteny analysis, and PPI of *PvGATA* gene family

**Fig. 3:**
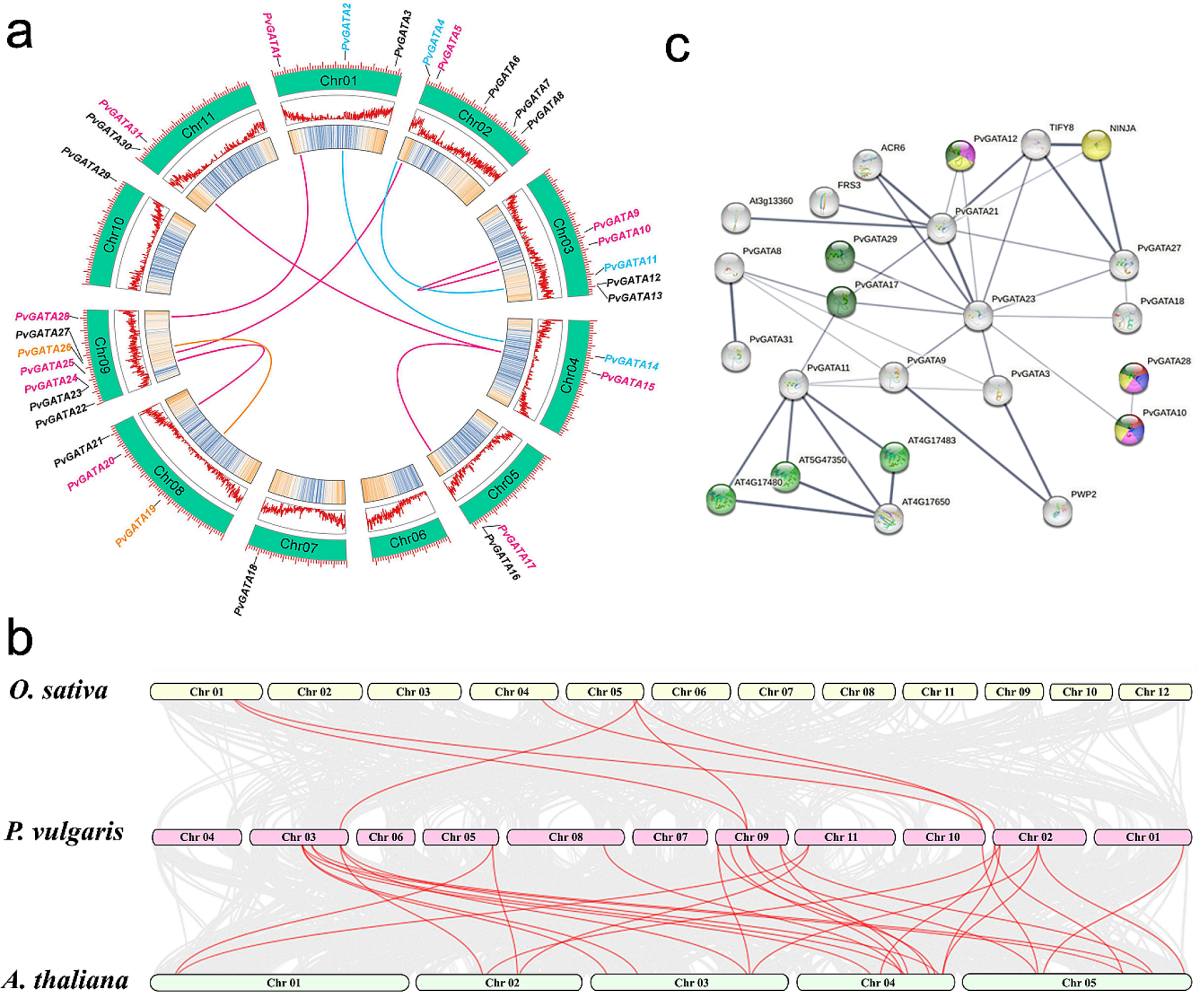
a: Chromosomal localization and synteny analysis of PvGATA proteins in the common bean genome. Genes IDs in black indicate the absence of collinearity, genes and lines colored in magenta indicate dispersed duplication; cyan indicates whole genome duplication, and golden colored lines indicate transposed duplicated pairs. The two rings in the center represent the chromosome’s gene density. b. Collinearity relationship analysis between *P. vulgaris* to *O. sativa* and *A. thaliana*. Gray lines indicate all synteny blocks found between the genomes of the species, and red lines indicate the gene pairs with duplicated events. c. Protein-protein interaction analyses were performed on the String web tool. The thickness of the lines represents the reliability of the results. The various functional associations are represented by different colors, including yellow for the regulation of shoot development, red for the negative regulation of gibberellic acid-mediated signaling pathway, blue for the negative regulation of seed germination, purple for the regulation of flower development, and green for positive regulation of nitrogen compound metabolic process

To understand the chromosomal distribution of the common bean *GATA* genes, we physically mapped the locations of the 31 *PvGATA* genes on the common bean genome (Fig. 3a). This investigation revealed a non-uniform distribution pattern across the chromosomes. Chr09 has the highest number of *PvGATA* genes (7), followed by Chr02 and Chr03, each containing 5 *PvGATA* genes. *PvGATA* genes were located on Chr01 and Chr08, with three genes on each chromosome. Chr04, Chr07, and Chr11 each contained 2 *PvGATA* genes, while the minimum number of 1 *PvGATA* gene was found on Chr05 and Chr10.

In our current study, we utilized the MCScanX method to delve into the phenomenon of gene duplication within the *PvGATA* gene family. Our analysis pinpointed nine distinct gene pairs that exhibited clear signs of duplication events. Out of these, six gene pairs were identified as undergoing dispersed duplication. In comparison, two gene pairs were linked to whole genome duplication, and a singular gene pair appeared to have undergone a transposed gene duplication event. To gain a deeper understanding of the evolutionary dynamics and selective pressures shaping the *PvGATA* genes, we diligently computed the substitution rate ratio Ka/Ks for the entire set of 31 *GATA* genes residing within the common bean genome. These calculations, meticulously presented in Table 2. consistently yielded Ka/Ks ratios below one, spanning a range from 0.381 to 0.126. Notably, two gene pairs, *PvGATA4-PvGATA11* and *PvGATA1-PvGATA28*, emerged with the highest and lowest Ka/Ks ratios, respectively. These findings collectively suggest a pronounced trend of purifying selection acting upon the *PvGATA* gene pairs throughout their evolutionary history. Such selective pressures underscore the crucial roles played by these gene pairs in preserving the conserved structural characteristics inherent to the *PvGATA* gene family.

**Table 2:** Inter-specific gene duplication analysis of *PvGATAs*

Gene1	Gene2	Identity (%)	Ka	Ks	Ka/Ks	Duplicated Type	Effective Length	MYA
*PvGATA1*	*PvGATA28*	46	0.628	4.968	0.126	dispersed	792	38.216
*PvGATA2*	*PvGATA14*	65	0.162	0.733	0.220	WGD	729	5.640
*PvGATA4*	*PvGATA11*	70	0.199	0.521	0.381	WGD	1605	4.009
*PvGATA5*	*PvGATA25*	72	1.059	3.165	0.335	dispersed	621	24.344
*PvGATA10*	*PvGATA9*	83	0.646	3.312	0.195	dispersed	468	25.479
*PvGATA16*	*PvGATA15*	82	0.653	3.419	0.191	dispersed	906	26.298
*PvGATA19*	*PvGATA26*	55	0.252	1.250	0.202	transposed	903	9.613
*PvGATA20*	*PvGATA24*	71	0.184	0.656	0.281	dispersed	813	5.045
*PvGATA31*	*PvGATA15*	82	0.589	3.180	0.185	dispersed	810	24.458

Ka, non-synonymous substitution per synonymous; ks, synonymous substitution per synonymous; WGD, Whole Genome Duplication; Mya, Million Years Ago

Based on protein-protein interaction (PPI) analyses, several GATA proteins, including PvGATA10, PvGATA12, PvGATA17, PvGATA28, and PvGATA29, have been found to play positive roles in the nitrogen compound metabolic process. On the other hand, PvGATA21 interacts with TIFY8 and NINJA, which are negative regulators of jasmonic acid signaling. PvGATA10 and PvGATA28 have been observed to have a negative role in the gibberellic acid-mediated signaling pathway and seed germination. Furthermore, PvGATA10, PvGATA12, and PvGATA28 are involved in shoot system development and flower development. These findings illuminate the multifaceted roles played by PvGATA proteins and their potential implications in various physiological processes within common bean plants.

### *Cis*-Regulatory elements in the promoter region of *PvGATA*

**Fig. 4:**
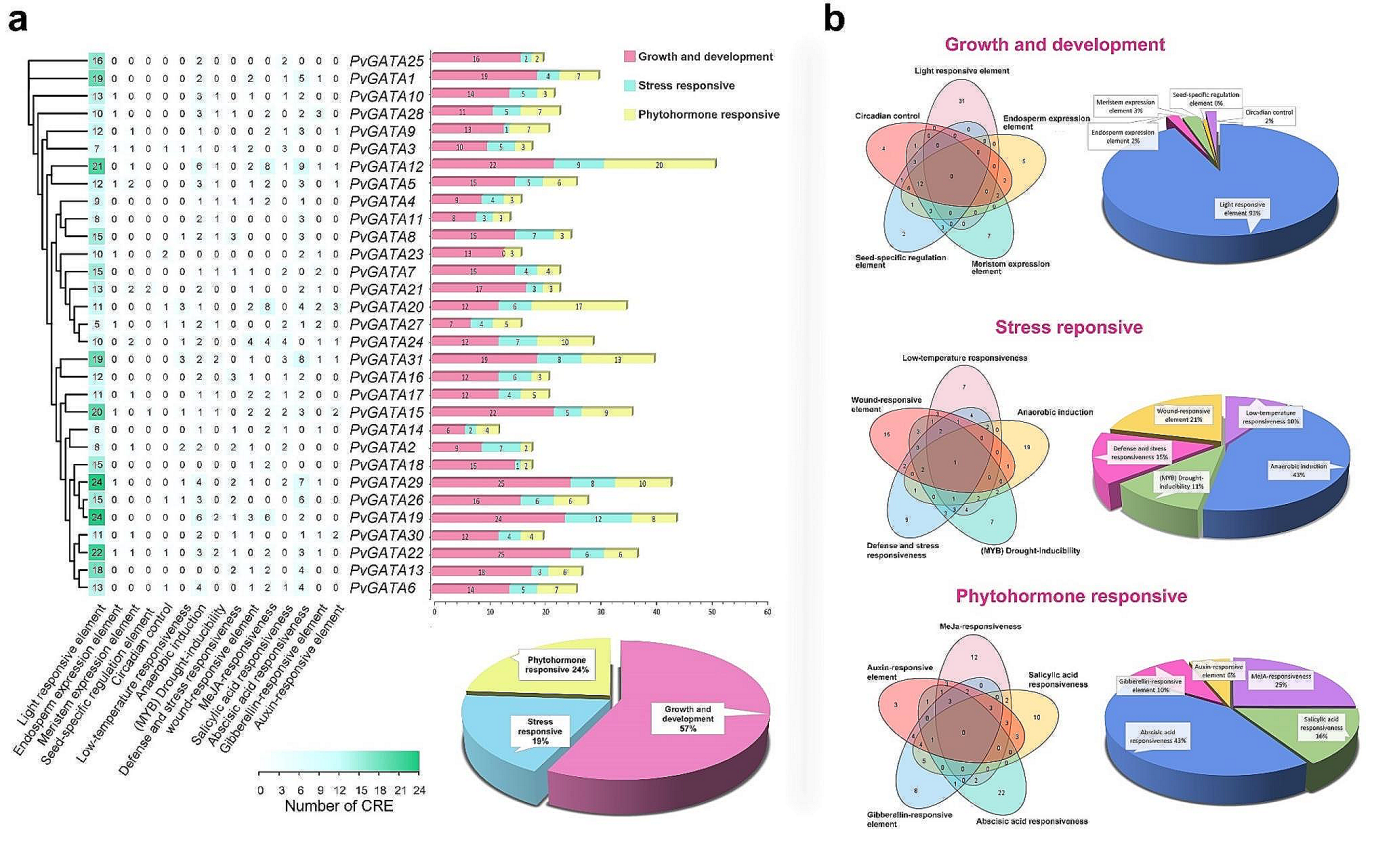
Analysis of *cis*-regulatory elements (CREs) in the putative promoter region of *PvGATA* genes using the PlantCARE database. (**a**) The number of predicted CREs located in the 2000 bp upstream of the *PvGATA* genes and the distribution of the three categories of CREs among the members of the *PvGATA* gene family. (**b**) Venn diagram and a pie chart showing the distribution of different functional categories of CREs identified in the *PvGATA* promoter region

To predict and analyze the promoter *cis*-regulatory elements (CREs) in *PvGATAs*, we retrieved the 2000 bp nucleotide sequences upstream of these genes. Subsequently, we submitted them for analysis using the PlantCARE database. The outcome revealed a total of 799 CREs distributed across all *PvGATAs*, with *PvGATA12* boasting the most substantial count at 51, while *PvGATA14* exhibited the lowest with 12 CREs. These *cis*-regulatory elements were then meticulously categorized into three distinct groups: Growth and Development regulatory elements, Stress-responsive elements, and Phytohormone-responsive elements, as visually represented in Fig. 4b and detailed in Table S2.

(i) Growth and development regulatory elements had the largest share of CRE in the promoter region of *PvGATA*, with a total of 457 elements and 57.20% of total CREs. All the subfamilies in *PvGATA* genes carried elements from this category except subfamily II, which only had elements associated with light responsiveness. Within this category, CREs light responsiveness (Box 4, LAMP element, GATA-motif, ATCT-motif, etc.) was heavily abundant, totaling 424 elements and 93% of the category’s total elements. Other elements in this category included meristem expression elements (CAT-box) with 13 elements (3%), endosperm expression (GCN4_motif) with nine elements (2%), circadian control with eight elements (2%) present in all *PvGATA* subfamilies except subfamily II and III, and seed-specific elements with three elements present in the promoters of *PvGATA15* and *PvGATA21* only.
(ii) The second category is stress-responsive CREs. A total of 151 (18.9%) CREs were predicted in the promoter region of *PvGATAs*. At least one element was present in all the PvGATA TFs except in *PvGATA23*. The stress-responsive category includes anaerobic induction elements (ARE), which had the most elements, with 65 (43%) predicted, followed by wound-responsive elements (WRE) with 32 elements (21%), defense and stress responsiveness with 22 elements (15%), (MYB) drought inducibility elements with 17 elements (11%), and low-temperature responsiveness (LTR) with the least number of elements, only 15 (10%).
(iii) The third category of CREs identified in the promoter region of *PvGATAs* is related to phytohormone response, with a total of 191 (23.9%) predicted CREs. The most common phytohormone-responsive element is the abscisic acid responsiveness element, with 82 (43%) CREs, followed by methyl jasmonate (MeJA)-responsive elements with 48 (25%) elements, salicylic acid-responsive elements with 30 (16%) CREs, gibberellin-responsive elements with 19 (10%) CREs, and auxin-responsive elements with the least number of CREs, only 12 (6%). Interestingly, subfamily II lacks CREs associated with gibberellin, auxin, and salicylic acid-responsive elements, suggesting the absence of these genes in the hormone regulation network.

### Functional GO annotation of PvGATA TFs

**Fig. 5:**
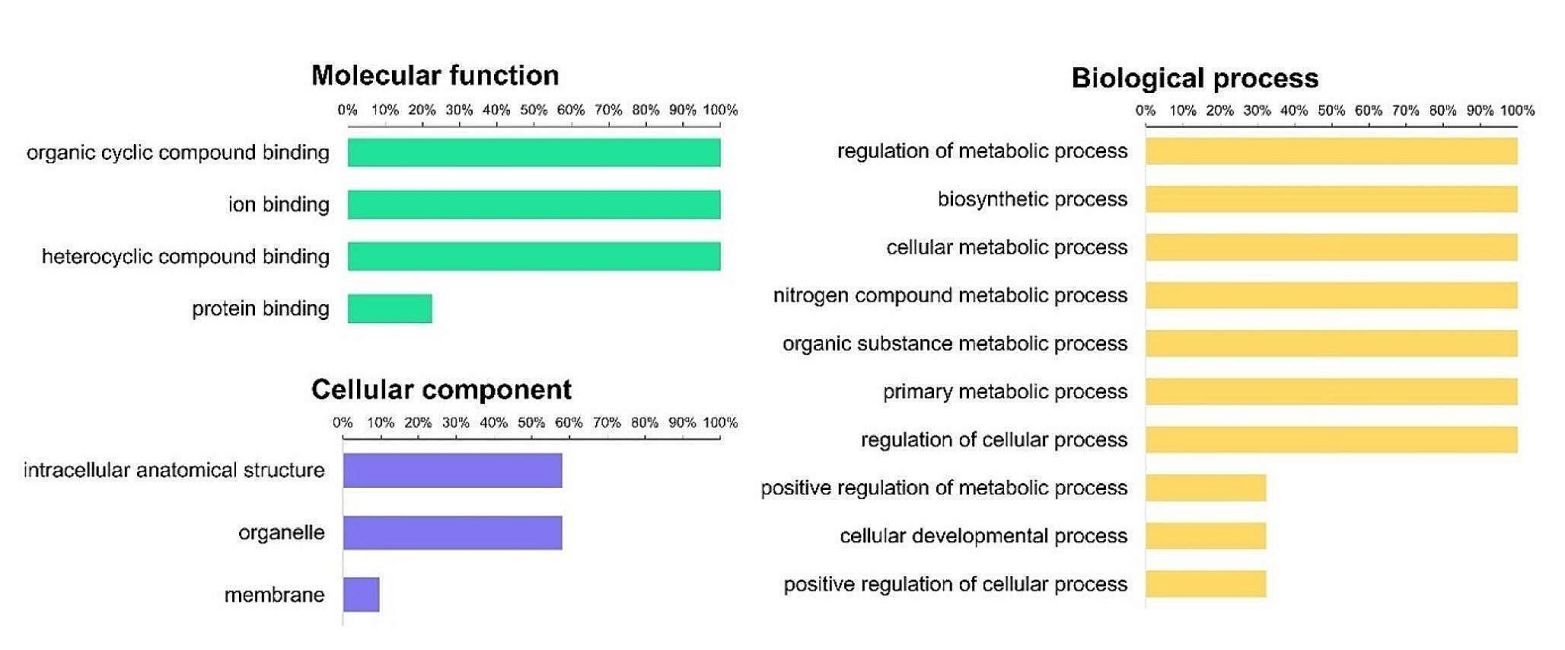
Gene ontology (GO) analysis of the *PvGATA* gene family using Blast2GO software. The distribution of GO annotations was determined for the genome-wide *PvGATA* gene family. The bar graph shows the percentage of *PvGATA* sequences assigned to different biological processes based on GO annotations, including molecular function, cellular component, and biological process

The functional annotation of PvGATA TFs was analyzed using the Blast2Go plugin in the OmicsBox software. The analysis revealed 17 gene ontology annotations in all *PvGATA*, which were categorized into three ontologies: molecular function, biological process, and cellular component (Fig. 5, Table S3). The molecular functions of *PvGATAs* were associated with protein, ion, metal, and DNA binding activities, which is consistent with the known association of GATA TFs with DNA binding activities. The cellular components of *PvGATAs* were predominantly located in the intercellular space, membrane, and nucleus, which underscores their importance in the development of common bean plants. Under biological process annotations, all *PvGATAs* were potentially involved in regulating metabolic processes such as nitrogen, organic compounds, and primary metabolites. Some PvGATA TFs were also associated with the positive regulation of cellular processes, as well as the development and regulation of cellular processes. Overall, these findings highlight the potential functions of *PvGATAs* in the development of common bean plants.

### Expression analysis of PvGATA TFs on subcellular levels, organ, and stress conditions

**Fig. 6:**
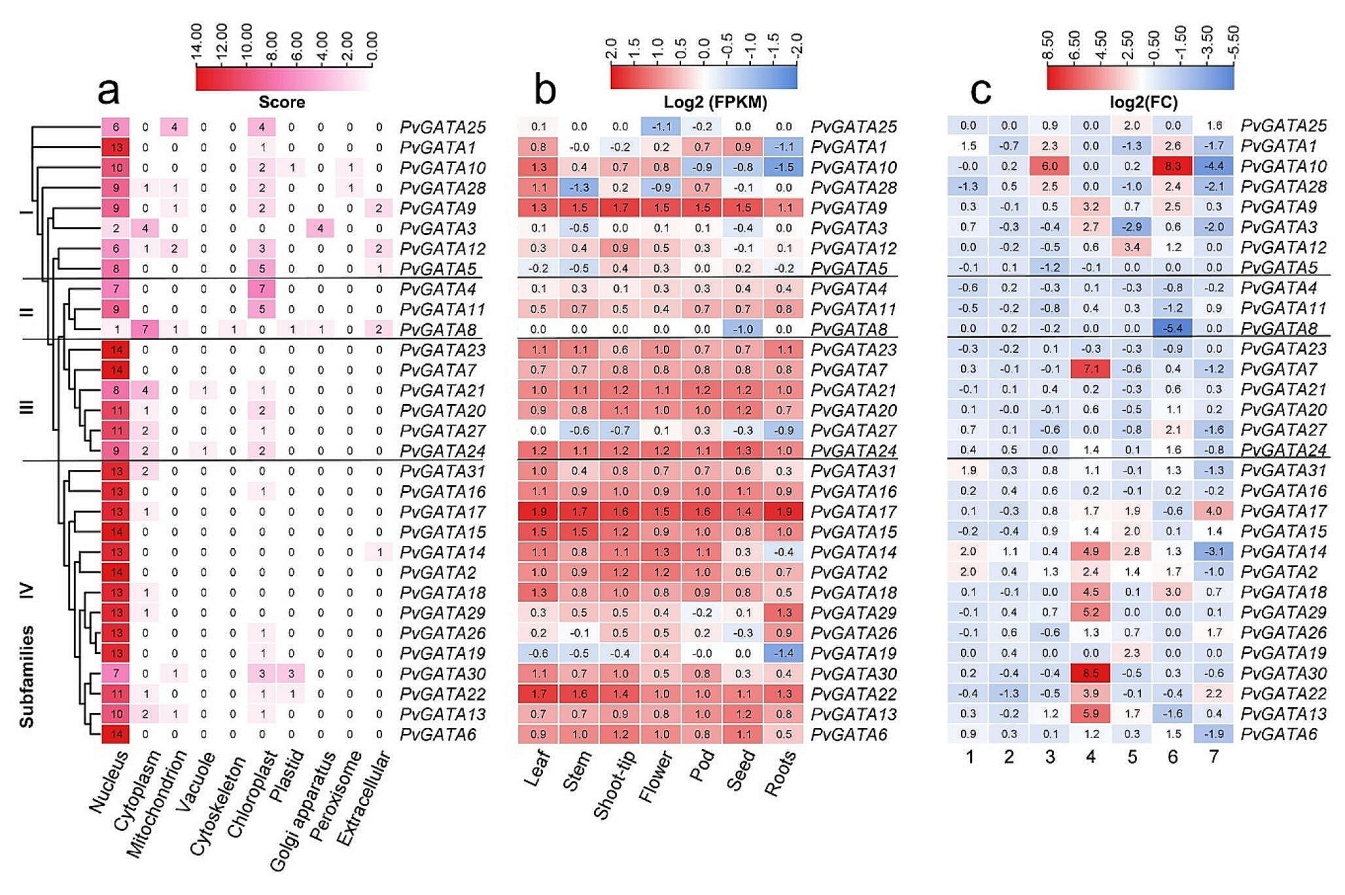
Heatmaps were generated to examine the expression patterns of PvGATA TFs under various cellular compartments, developmental stages, and stress conditions. The heatmaps were constructed and visualized using TBTools software. Black lines are used to differentiate between the subfamilies. (**a**) the sub-cellular localization of PvGATA proteins was predicted using the WoLF PSORT web tool. (**b**) The tissue-specific expression profiles of PvGATA at different developmental stages of the common bean plant were analyzed using publicly available transcriptome data (PRJNA210619) and displayed in a heatmap. The normalized fragments per kilobase of transcript per million fragments (FPKM) values were transformed by Log2(FPKM). (**c**) Expression analysis of *PvGATA* transcripts on different biotic and abiotic stress conditions using RNA-seq data publicly available. 1 and 2 samples were collected from the leaves and roots of a salt-tolerant vs. salt-sensitive cultivar under salt-stress conditions. (PRJNA656794). 3, Samples from lower hypocotyl under salt stress after 12 h vs. control (PRJNA691982). 4 drought stress of resistant cultivar vs. control at 150 min after treatment. 5, Sensitive cultivar under drought stress vs. control (PRJNA508605). 6, fungal plant pathogen infected vs. non-infected samples of the resistant cultivar (PRJNA574280). 7, low-temperature stress of resistant common bean plant vs. control treatment (PRJNA793687).

In order to investigate the subcellular localization of the PvGATA proteins, we utilized the WoLF PSORT online database. Our results revealed that all PvGATA proteins, except PvGATA3 and PvGATA8, were predominantly localized in the nucleus (Fig. 6a, Table S4). Which suggests their function as transcription factor. To be precise, members of subfamily IV, such as PvGATA2, PvGATA6, and PvGATA15, and subfamily III, such as PvGATA23 and PvGATA7, were predicted to be 100% localized in the nucleus. Furthermore, a subset of PvGATA proteins was predicted to localize to other subcellular organelles. For instance, members of subfamily II, including PvGATA4 and PvGATA11, were found to be significantly present in the chloroplast, PvGATA8 was predicted to be localized in the cytoplasm and extracellular organelles. In subfamily I, PvGATA28 was predicted to be present predominantly in nucleus and also some traces found in chloroplast, peroxisome, cytoplasm and mitochondria.

We investigated the expression profile of GATA TFs in various tissues of the common bean plant using publicly available transcriptome data (O’Rourke et al. 2014). The tissues analyzed included leaf, stem, shoot tip, apical meristem, young flower, young flower buds, seeds, and whole roots with root tips. As shown in Fig. 6, we observed significant differences in the expression patterns of *PvGATA* transcripts among the subfamilies (Table S4). In subfamily I, almost all *PvGATA* genes, except for *PvGATA5*, were highly expressed in vegetative leaf tissues. In contrast, *PvGATA9* was highly expressed in all tissues analyzed, with the highest expression in shoot tips, suggesting its crucial role in tissue development. The expression profile of subfamily I members was considerably downregulated or had no expression in root and root-tip tissues, except for *PvGATA9*. In subfamily II, gene expression was moderate and less than 0.8, indicating their subordinate role in organ development. In subfamily III, a uniform preferential expression pattern was noted from all members, except for *PvGATA27*, which was negatively regulated on roots, stem, and shoot tips. Gene members in subfamily IV were preferentially expressed in vegetative and flower tissues. Among these genes, *PvGATA17* was highly expressed in all tissues, followed by *PvGATA15/22. PvGATA19* had the least expression profile among the gene members, especially in roots. These findings reveal a unique and tissue-specific expression pattern of PvGATA TFs, which suggests their functional specialization in various tissues and developmental processes.

Based on previously generated RNA-seq data, we analyzed the expression profile of candidate *PvGATA* gene members in response to various abiotic and biotic stress conditions (Fig. 6c, Table S4). Significant levels of positive and negative expression were observed among the *PvGATA* genes. Specifically, in both leaf and root tissues of common bean cultivars treated with saline water, *PvGATA* genes were preferentially expressed. Subfamily IV members showed the highest positive expression in leaf tissues, while subfamily II members were down-regulated. Notably, *PvGATA* genes displayed moderate expression in root tissues of resistant cultivars. In contrast, subfamily I members were upregulated in lower hypocotyl tissues of salt-treated cultivars compared to the control. Similar expression patterns were observed for *PvGATA1/28*, whereas *PvGATA3/12/5* showed relatively lower expression levels. Subfamily II and III members exhibited relatively low expression, while subfamily IV had a moderately positive expression profile. Under dehydration stress in the drought-resistant cultivar Perola, subfamily IV members were predominantly upregulated, with *PvGATA30/22/13/14/18* exhibiting the largest increase in up-regulation level. A similar expression pattern was observed for gene members of subfamily I and III, such as *PvGATA9/3* and *PvGATA7*, albeit to a lesser extent. In contrast, subfamily II gene members showed lower expression levels than all other genes. In drought-sensitive cultivars, a reverse expression pattern was observed across all genes, with *PvGATA30/7/3/18/29/22* being negatively expressed. This result suggests the potential importance of these genes in regulating resistance to drought stress in common bean plants. When exposed to fungal stress (*Sclerotinia sclerotiorum*), subfamily I members showed a significant increase in expression, with the highest expression value observed for *PvGATA10*. Subfamily III and IV members also exhibited increased expression levels, while the lowest expression levels were observed for *PvGATA8*, a member of subfamily II. *PvGATAs* displayed relatively low expression levels in response to cold stress across all subfamilies, with increased expression observed for *PvGATA17/22*. The most negatively expressed genes were *PvGATA10/14/28/3*, suggesting a potentially lesser role for *PvGATA* in modulating resistance to cold stress. In short, the expression patterns of *PvGATA* genes in different stress conditions suggest potential functional differences among the subfamilies.

**Fig. 7:**
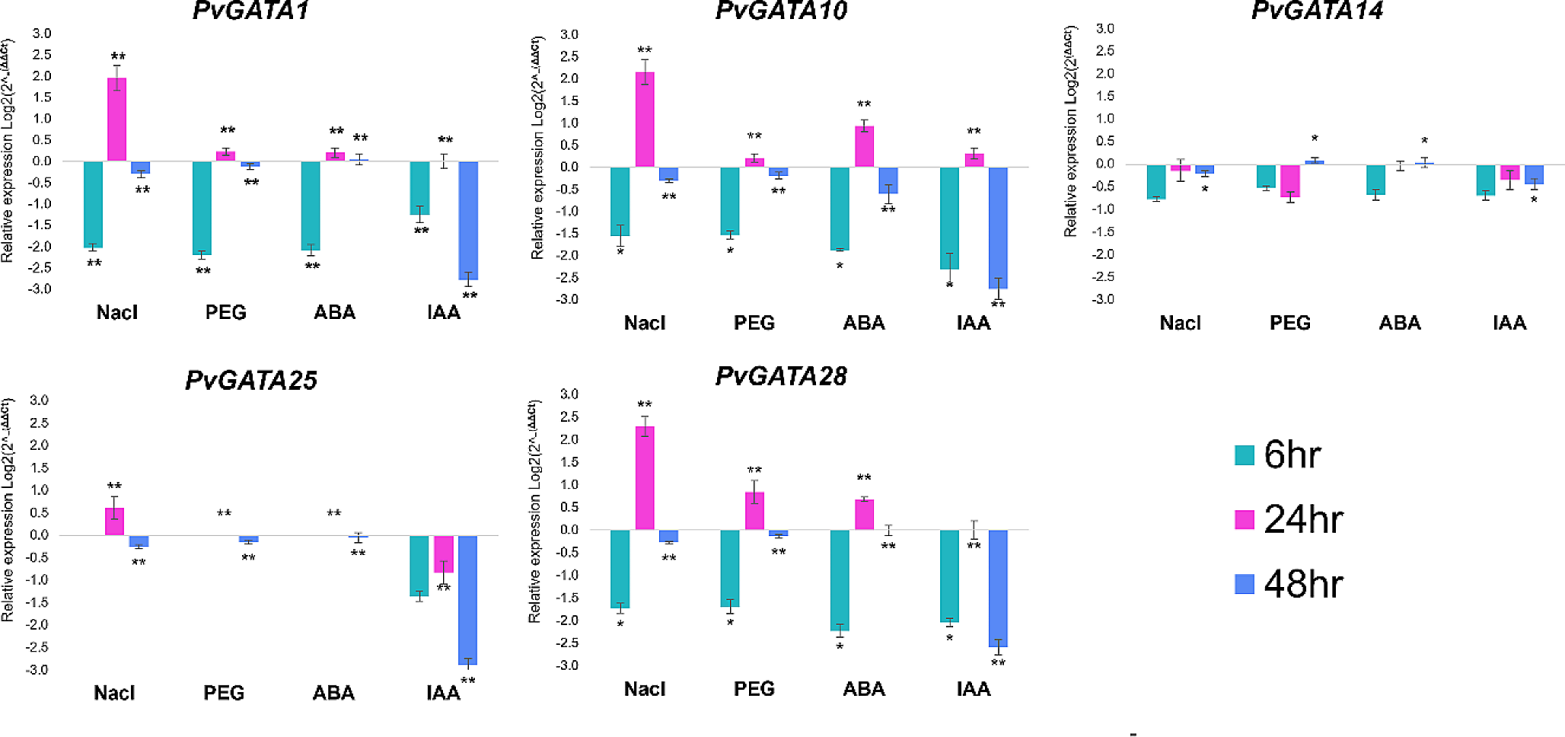
qRT-PCR expression profile analysis of five candidate *PvGATA* genes (*PvGATA1*, *PvGATA10*, *PvGATA14*, *PvGATA25*, and *PvGATA28*) under four different abiotic and phytohormone stress conditions, including Salinity stress (NaCl), Drought stress (PEG), Abscisic acid (ABA), and Indole acetic acid (IAA). Samples were collected from root tissues at three time points after treatment: 6-hour, 12-hour, and 48-hour. The experiments were performed independently with three replicates, and the error bars represent the standard deviation of three replicates. Asterisks indicate significant differences in transcript levels compared with the blank control. *P < 0.05, **P < 0.001

To confirm the expression profiles of selected *PvGATA* genes to the public RNA-data, we performed expression analysis using the qRT-PCR with RNA extracted from roots subjected to different abiotic and phytohormone treatments (Fig. 7). Based on the expression levels of *PvGATAs* from previously analyzed RNA-Seq data (Fig. 6c), we selected five *PvGATA* genes with the highest expression levels as candidate genes for further analysis. Under saline treatment, all analyzed genes were negatively expressed at 6 h compared to the control. At 24 h, *PvGATA1*, *PvGATA10*, and *PvGATA28* were highly upregulated, and *PvGATA25* was also positively expressed, albeit at a relatively lower level. At 48 h, the expression levels were negative.

Similarly, under drought treatment, all five genes were significantly down-regulated at 6 h but peaked at 24 h and showed low expression levels at 48 h. Under phytohormone treatments with ABA (Abscisic acid) and IAA (Indole acetic acid), *PvGATAs* were generally negatively expressed, with a few exceptions. *PvGATA1*, *PvGATA10*, and *PvGATA28* were significantly upregulated under ABA treatment compared to the control, and *PvGATA10* was upregulated significantly under ABA and IAA treatment at 24 h. Our results support findings from our previous RNA-seq analysis (Fig. 6c) and suggest that *PvGATA1/10/25/28* may play crucial roles in regulating plant resistance against salt and drought stress at 24 h. Additionally, these GATA genes may be involved in the phytohormone-mediated stress signaling pathways in the common bean plant.

### Subcellular localization of *PvGATA28* in *N. benthamiana*

**Fig. 8:**
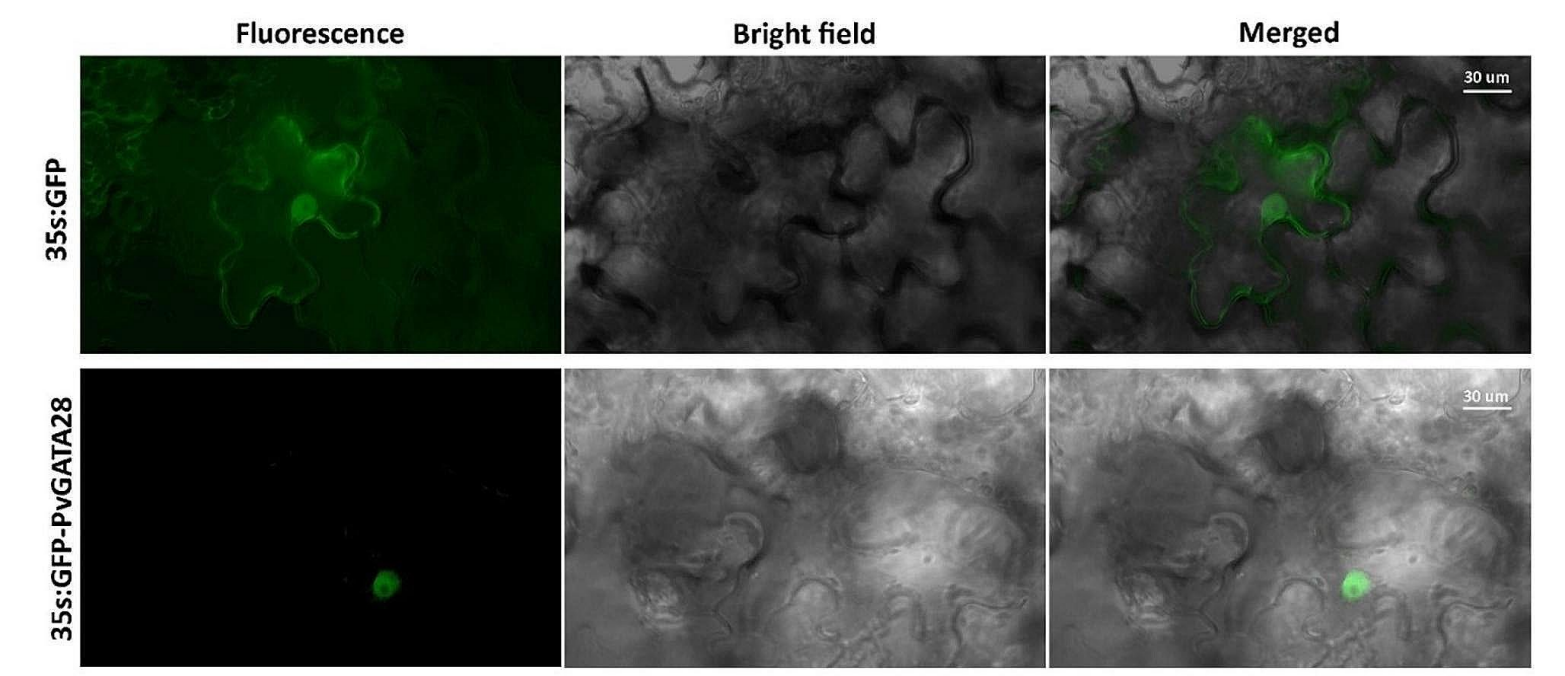
Localization of *PvGATA28* in *Nicotiana benthamiana* leaf epidermal cells using transient expression. Confocal microscopy reveals that the GFP-PvGATA28 fusion protein localizes to the nucleus, whereas the control GFP protein is present in both the cellular membrane and nucleus. Merged images combine bright field and fluorescence signals. Scale bar = 30 μm

To assess the specific subcellular localization of *PvGATA28*, we constructed the 35 S:GFP-PvGATA28 fusion protein, incorporating the coding sequence of *PvGATA28* linked to the C-terminus of the GFP reporter gene, all under the control of the *CaMV35S* promoter. A construct containing solely GFP served as our negative control. Through confocal microscopy analysis, it was observed that the 35 S:GFP-PvGATA28 fusion protein was exclusively localized within the nucleus, contrasting with the control 35 S:GFP protein, which was distributed throughout both the cytoplasm and membrane in the leaf epidermal cells. This evidence indicates that PvGATA28 proteins are localizes specifically to the nucleus (Fig. 8).

### Over expression of *PvGATA28* in *N. benthamiana*

In this study, the coding sequence of *PvGATA28* was successfully cloned from the cDNA of *P. vulgaris* and into a plant expression vector controlled by a constitutive double *CaMV  35S* promoter. The cloned sequence was then introduced into *N. benthamiana* leaves through in-vitro transformation using *Agrobacterium tumefaciens* strain GV3101. To ensure the accuracy of the cloning process, Sanger sequencing was performed prior to the transformation into tobacco leaves. Two transgenic lines (OE line 1 and OE line 2) were selected based on their performance following stress treatments for subsequent analysis, along with the wild-type line.

**Fig. 9:**
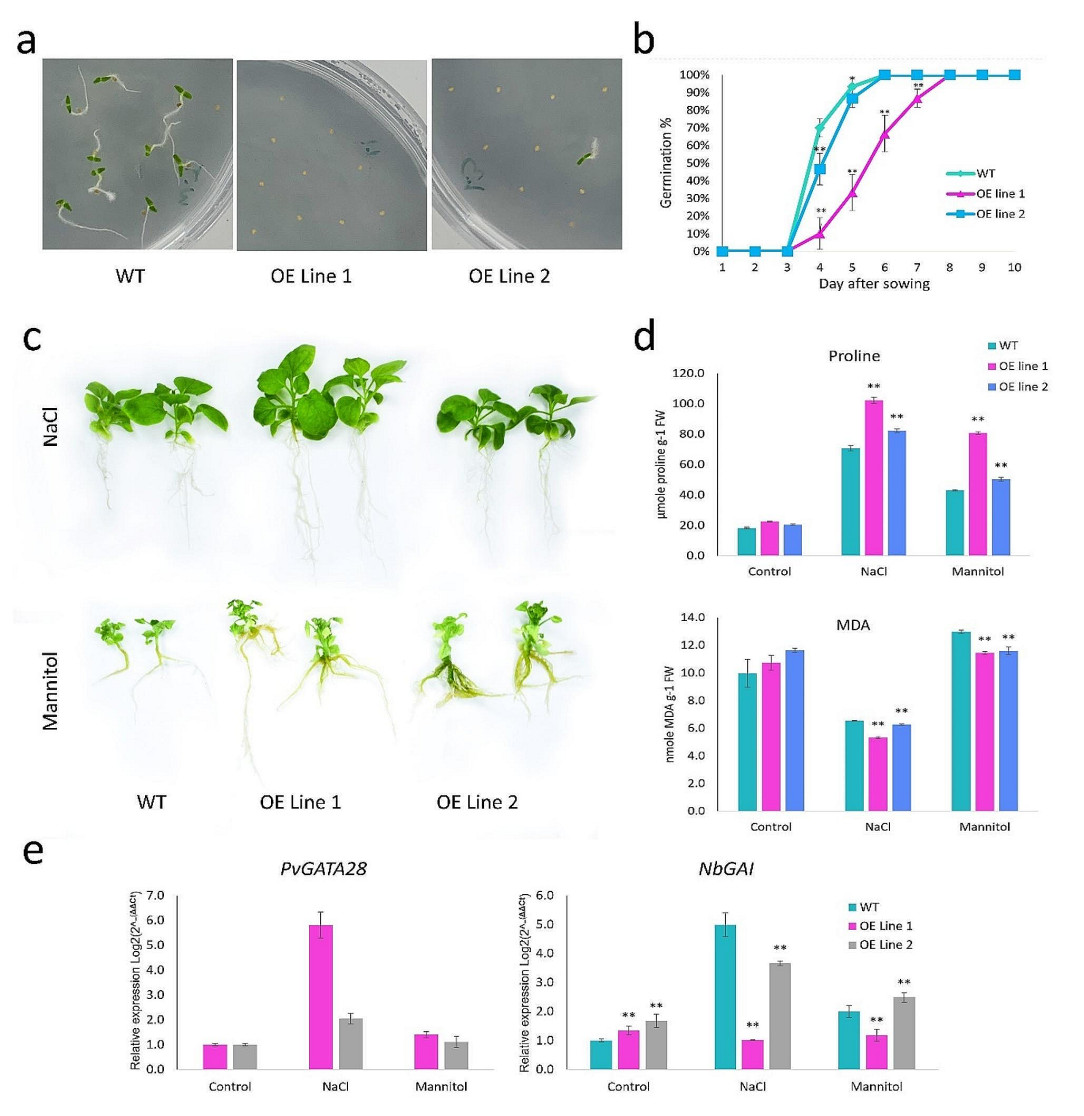
Effect of *PvGATA28* overexpression in N. benthamiana on physiological aspects of plant’s response to abiotic stress. (**a**) *PvGATA28* overexpression significantly influenced seed germination, as observed by daily monitoring of surface-sterilized seeds on half MS media over ten days. (**b**) The graph depicts the germination percentage over days in transgenic lines compared to the wild type. (**c**) Under invitro conditions, transgenic lines exhibited enhanced tolerance to NaCl, and mannitol stress compared to wild-type photographs captured four weeks post-sowing. (**d**) Abiotic stress effects on MDA and proline content were assessed, revealing altered stress responses in transgenic and wild-type lines. (**e**) Expression profiling of *PvGATA28* and NbGAI was performed under NaCl and mannitol stress in transgenic and wild-type lines. ** indicates a significant difference at *P* ≤ 0.001; error bars represent variation among three replicates

To assess the effect of *PvGATA28* on the germination rate of *N. benthamiana* seeds, T1 transgenic lines 1 and 2, along with the wild-type line, were subjected to surface sterilization before being sown on Petri plates containing a half MS solution. The germination rate was monitored and recorded over ten days. The investigation findings revealed a significant difference between the *PvGATA28* overexpressing lines (lines 1 and 2) and the wild-type line (Fig. 9a). Remarkable discrepancies were observed on the fourth and fifth days following seed sowing (Fig. 9b). During these specific time points, the wild-type lines displayed 70% and 93% germination rates on days 4 and 5, respectively. Interestingly, OE line 1 exhibited significantly lower germination rates, registering only 10% and 33% on the same days.

In contrast, in the case of OE line 2, germination rates of 47% and 87% were documented for days 4 and 5, respectively. These results highlight how the *PvGATA28* affects the germination rate of transgenic *N. benthamiana* seeds. The lower germination rates in *PvGATA28*-overexpressing lines suggest that *PvGATA28* likely plays a vital role in controlling seed germination and dormancy.

To evaluate stress tolerance in transgenic *N. benthamiana* lines, the selected plant lines were exposed to salt and drought stress conditions in vitro. This was achieved by supplementing the MS media with 150 mM NaCl to represent salt stress and 150 mM mannitol to simulate drought stress (Fig. 9c). The plant samples were collected six weeks after planting, and their physiological responses were analyzed to assess stress tolerance. Specifically, we measured the malondialdehyde (MDA) levels and proline content in the plant samples (Fig. 9d). The results of this study indicated a significant impact of proline content on the transgenic lines compared to the wild-type control line under both salt and drought stress treatments. Notably, under salt stress, OE line 2 exhibited a proline content of 102 (µmole proline g-1 FW), whereas the wild-type plants showed only a proline content of only 70. Similarly, under drought stress conditions, OE line 2 had a proline content of 80, while the wild-type plants exhibited a proline content of merely 43. These findings underscore the enhanced stress tolerance of the transgenic *PvGATA28* expressing lines compared to the wild-type plants.

The abiotic stress conditions, including salt and drought, notably influenced the levels of MDA content. During salt stress, OE line 2 displayed an MDA content of 5 (nmole MDA g-1 FW), followed by OE line 5 with 6.2, while the wild-type line registered 6.5. Similarly, under drought stress conditions, OE lines 1 and 2 demonstrated MDA levels of 11.4 and 11.6 (nmole MDA g^-1 FW), respectively. In contrast, the wild-type lines exhibited a higher MDA content of 12.9. These findings emphasize the enhanced stress tolerance nature of the OE lines compared to the wild-type lines, underscoring the inherent tolerance of *PvGATA28* to abiotic stressors.

In addition, qRT-PCR was performed to measure the expression levels of *PvGATA28* in transgenic lines under salt and drought stress conditions (Fig. 9e). In samples exposed to NaCl treatment, OE line 1 had the highest expression level, up to 5.7-fold compared to the control-treated OE line 1 samples. Whereas OE line 2 recorded two-fold increase compared to the control treated OE line2. Under Mannitol treatment, OE lines 1 and 2 demonstrated MDA levels of 11.4 and 11.6 (nmole MDA g^-1 FW) respectively. These findings suggest that the expression level of *PvGATA28* in transgenic lines could regulate the plant’s tolerance to salt and drought conditions. To further investigate the effect of *PvGATA28* overexpression on the gibberellic acid biosynthesis in *N. benthamiana*, qRT-PCR was performed on transgenic lines and wild type counterparts. For this study, *GIBBERELLIC ACID INSENSITIVE* (*NbGAI*, a *DELLA* domain-containing gene) was selected to investigate the regulatory effect of *PvGATA* under control and stress conditions. Under control conditions, OE line 1 was seen upregulated to 1.35-fold compared to the wild type samples. On the other hand, OE line 2 was mildly higher with 1.7-fold compared to wild type. Interestingly, under salt stress conditions, expression levels of *NbGAI* were higher in wild type samples and compared to OE lines 1 and 2. However, under mannitol treatment, OE line 1 was observed to have a significantly lower expression level than the wild type. In comparison, OE line 2 had a significantly higher expression level compared to wild type plants.

## Discussion

### Characteristics of the *GATA* gene family in *Phaseolus vulgaris*

The transcription factor GATA has been extensively studied in many plants for their diverse crucial biological processes in plants by regulating the expression of genes responsible for the development, stress, and hormonal signaling (An et al. 2019; Manzoor et al. 2021; Reyes et al. 2004; Wang et al. 2023; Zhao et al. 2021). The functional and molecular mechanism of GATA TFs in regulating abiotic stress tolerance has not yet been studied in common bean plants. Hence, this study analyzed the functional identity of the *GATA* gene family in the *Phaseolus vulgaris* genome under different abiotic and phytohormonal stress.

In this study, we systematically analyzed the *P. vulgaris GATA* gene family. We identified 31 GATA TFs, a number close to that found in *Arabidopsis thaliana* (29), *Oryza sativa* (28) (Reyes et al. 2004), *Brachypodium distachyon* (27) (Guo et al. 2021), and *Capsicum tetragonum* (28) (Yu et al. 2021). More than those found in *Eucalyptus urophylla* (23) (Du et al. 2022) and *Prunus avium* (18) (Manzoor et al. 2021) and less than those found in *Triticum aestivum* (79) (Feng et al. 2022) and *Brassica napus* (96) (Zhu et al. 2020).

Gene duplication can occur in two ways; either through whole genome duplication (WGD) or, through smaller scale duplications, including, dispersed duplication and transposed duplication. These replication processes can result in the emergence of genes with different functions or drive functional divergence. In our investigation, we found that the collinearity between common bean and *Arabidopsis* was higher than in rice. This shows that the number and function of *GATAs* are closely associated with species type. Among the 31 *PvGATAs*, 18 genes were duplicated, including six dispersed duplicated pairs, two WGD pairs and one transposed duplicated pair. These results suggest the importance of gene duplication in contribution to *GATA* gene expansion in common bean and corresponds with previous findings in pepper, *Gossypium*, and apple (Chen et al. 2017; Yu et al. 2021; Zhang et al. 2019). This prevalence of genes has had an impact, on the evolution of *PvGATA* by enabling the emergence of new functions. For instance genes duplication were previously reported to have contributed to the development of structures, activation of disease resistance mechanisms and adaptation, to environmental stress conditions (Panchy et al. 2016). However, the impact of the scale of duplication on how genes evolve, and function is not yet fully understood, primarily due to our knowledge of protein function and the varying timing of duplication events (Guan et al. 2007; Roth et al. 2007).

In previous reports phylogenetic analysis of GATA TFs identified seven subfamilies according to their conserved motif structure. In *PvGATAs* we grouped the genes into four subfamilies which lacks subfamilies V, VI, and VII which were previous reported in rice (Reyes et al. 2004), suggesting the conserved structure and function of GATA TFs in plants. In our study, we identified eight, three, six and 13 members in each subfamily (subfamilies I. II, III, and IV) (Figs. 2 and 3). A close relation in motif conserved domain and exon: intron structures was observed among same subfamily members suggesting their close functionality, but there have yet been any reports on the functional differences among these subfamilies.

Most GATA TFs in plants contain a single zinc finger domain in their protein sequence, such as those found in *Arabidopsis*, rice, wheat, and grape (Feng et al. 2022; Reyes et al. 2004; Z. Zhang et al. 2018). A single GATA domain was also present in all PvGATA protein sequences, however, members of subfamily II contained additional domains like CCT and tify domains. Previous studies also observed this and suggest a key role in diverse biological functions. For example, in regulating embryo and flower development, stress tolerance, and different phytohormone signaling (Behringer and Schwechheimer 2015; Gupta et al. 2017; Richter et al. 2013).

GATA TFs were first predicted and identified in regulating light-associated genes and phytohormonal-regulated photomorphogenesis in *Arabidopsis* and *P. edulis* (Luo et al. 2010; Zhang et al. 2018). Hence, we predicted and analyzed the promoter *cis*-regulatory elements (CREs) in the 2000 bp upstream of *PvGATAs*. The results predicted a total of 799 CREs in all the *PvGATAs*. These CREs were categorized into three categories, namely growth and development regulatory elements, stress-responsive elements, and phytohormone-responsive elements. The growth and development regulatory elements had the largest share of CREs in the promoter region of *PvGATA* genes, with a total of 457 elements and 57.20% of total CREs. Within this category, CREs light responsiveness was heavily abundant, totaling 424 elements and 93% of the category’s total elements. This was similar to studies on cucumber and *Rosaceae sp* (Zhang et al. 2021a, b). Light profoundly influences various plant processes, including circadian rhythm, photosynthetic regulation, and development (Ma et al. 2010). As previously mentioned, GATA TFs may mitigate excessive light damage and enhance photosynthetic activity by promoting chloroplast development. This then increases the conversion of light energy into chemical energy and results in enhanced accumulation of carbohydrates (An et al. 2019; Dordas and Sioulas 2008). However, exploring this hypothesis can be done through further experiments involving excessive light stress in common bean plants. Other elements in this category included meristem expression elements, endosperm expression, circadian control, and seed-specific elements.

The second category is stress-responsive CREs, with 151 (18.9%) CREs predicted in the promoter region of *PvGATAs*. This category includes anaerobic induction elements, wound-responsive elements, defense and stress responsiveness, drought inducibility elements, and low-temperature responsiveness. Anaerobic induction elements were most abundant within this category, indicating the vital role of *GATAs* in environmental stress resistance in common bean plants. The third category of CREs identified in the promoter region of *PvGATAs* is related to phytohormone response, with a total of 191 (23.9%) predicted CREs. The most common phytohormone-responsive element is the abscisic acid responsiveness element, followed by methyl jasmonate (*MeJA*)-responsive elements, salicylic acid-responsive elements, gibberellin-responsive elements, and auxin-responsive elements. Interestingly, subfamily II lacks CREs associated with gibberellin, auxin, and salicylic acid-responsive elements, suggesting the absence of these genes in the hormone regulation network. The identification of CREs in the promoter region of *PvGATAs* provides insight into the regulation of gene expression, particularly in growth and development, stress response, and phytohormone signaling. This information can be used to study gene expression and regulation, which can aid in crop improvement and stress tolerance. Understanding the functions of these regulatory elements in *PvGATA* genes can help identify molecular targets for crop improvement and breeding.

The *GATA* gene family encodes TFs that regulate gene expression by recognizing a specific consensus sequence NGATAY (N = T or A; Y = G or A(Lowry and Atchley 2000). Nitrogen levels significantly impact plant growth and carbon uptake(GUERRIERI et al. 2011; Manzoor et al. 2021), with low levels generally benefiting these processes, while high levels may decrease water use efficiency (Lu et al. 2018). In our study, we used PPI analyses to determine the roles of specific PvGATA proteins. PvGATA10, PvGATA12, PvGATA17, PvGATA28, and PvGATA29 play positive roles in nitrogen compound metabolic processes. Overexpression of these proteins could increase nitrogen usage, potentially counteracting the negative effects of high nitrogen levels in the soil. In addition to their roles in nitrogen metabolism, we also observed interactions between some PvGATA proteins and plant hormone signaling pathways. Plant hormone signaling is essential for responding to biotic and abiotic stressors, and our findings suggest that PvGATA proteins may play a role in these responses. For example, some studies have linked reduced cytokinin signaling to increased drought tolerance (Liu et al. 2017; Nishiyama et al. 2013). In another study, the knockout of the gene that encodes the DELLA protein, a negative gibberellic acid signaling pathway regulator, has resulted in salt sensitivity (Achard et al. 2006). Conversely, overexpression of the gibberellic acid-insensitive-1 gene from *Arabidopsis* in Petunia has been linked to increased drought tolerance (Zhang et al. 2021). Therefore, it is understood that reduced gibberellic acid signaling leads to increased drought tolerance and decreased salinity tolerance. Our study identified *PvGATA10* and *PvGATA28* as negative regulators of the gibberellic acid signaling pathway, suggesting that overexpression of one of these genes could lead to increased drought tolerance. This finding supports the potential use of these genes in developing drought-tolerant plants. Overall, our study provides valuable insights into the roles of specific PvGATA proteins in nitrogen metabolism and plant hormone signaling, offering potential avenues for future research on plant stress responses and crop improvement.

### *PvGATA* regulates abiotic and biotic stress tolerance

The expression profile of candidate *PvGATA* gene members in response to various abiotic and biotic stress conditions has been analyzed based on previously generated RNA-seq data. The study revealed that *PvGATA* genes exhibited a significant level of positive and negative expression among the subfamilies in response to different stress conditions. The study also confirmed the expression profiles of selected *PvGATA* genes (*PvGATA1*, *PvGATA10*, *PvGATA14*, *PvGATA25*, and *PvGATA28*) using qRT-PCR with RNA extracted from common bean roots subjected to different abiotic and phytohormone treatments. The results were consistent with the previously analyzed RNA-Seq data, suggesting that *PvGATA1/10/25/28* may be crucial in regulating plant resistance against salt and drought stress. Similar results were also reported in Sweet potato, *IbGATA24* enhanced the hormonal signaling pathways and scavenging of the reactive oxygen species (ROS) and hence regulating plant stress tolerance (Zhu et al. 2022). In wheat, qRT-PCR expression analysis of *TaGATA* under NaCl, PEG, and ABA stress showed significant differences indicating regulatory function of *GATA* under these treatments (Du et al. 2023). Earlier study demonstrated the involvement of two *GATA* related genes in Arabidopsis, *GNC* and *GNL/CGA1*, in regulating auxin and gibberellin signaling (Richter et al. 2013). In our study the expression levels of *PvGATA* under IAA had significant reduction. Additionally, these *GATA* genes may be involved in the phytohormone-mediated stress signaling pathways in the common bean plant. The subcellular localization analysis, showed that PvGATA proteins were predominantly localized in the nucleus, this suggests its functioning in transcription factor activity, a similar result was reported in sweet potato, grape and tomato (Z. Zhang et al. 2018; Zhao et al. 2021; Zhu et al. 2022).

Some studies have shown that overexpression of certain *GATAs* like *GATA12* negatively regulates seed germination by binding downstream of the genes encoding *DELLA* proteins, which are negative regulators of gibberellic acid signaling (Ravindran, Verma, Stamm, & Kumar, 2017). However, another study in tomato plants has shown that the *SIGATA27* protein plays a positive role in seed germination (Richter et al. 2010; Wang et al. 2023). In our study, *PvGATA28* overexpressed transgenic lines have shown later germination than WT lines. We investigated and compared the expression levels of *GAI*, which is a *DELLA* protein-encoding gene, between OE lines and WT lines to verify these findings at the molecular level (Fig. 8e). Physiological and molecular analyses have shown that overexpression of *PvGATA28* in *Nicotiana benthamiana* results in delayed germination by decreasing gibberellic acid signaling.

GATA transcription factor has been previously reported to play a positive role in enhanced stress regulation in plants. However, such analysis has not yet been demonstrated in *P. vulgaris*. To further investigate whether *PvGATA* is involved in the stress responsiveness in common bean, we conducted an abiotic stress analysis on the transgenic *N. benthamiana* lines overexpressing *PvGATA*. This analysis included the application of salt and drought stress treatments, along with Proline and MDA analyses. The lower MDA levels observed indicate reduced electrolyte leakage and membrane damage, suggesting mitigation of excess reactive oxygen species (ROS) production. Conversely, higher proline levels suggest that *PvGATA28* transgenic lines exhibit enhanced oxidation resistance and ROS scavenging capabilities (Fig. 9d). The observed upregulation of *PvGATA28* expression in transgenic lines compared to the wild type under salt treatment suggests a potential role for *PvGATA28* in salt tolerance, potentially more pronounced than its involvement in drought treatment (Fig. 9e). This pattern aligns with findings in rice, where overexpression of *OsGATA8* enhanced plant tolerance to drought and salt treatments (Gupta et al. 2017). Similarly, in tomato, *SlGATA17* overexpressing lines demonstrated improved performance under drought and salt stress treatments (Zhao et al. 2021). Conversely, the knockout of the same *SlGATA17* was shown to reduce tolerance to salt stress by suppressing ROS production (Wang et al. 2023).

The subcellular localization analysis, showed that all PvGATA proteins were predominantly localized in the nucleus, this suggests its functioning in transcription factor activity, a similar result was reported in sweet potato, grape and tomato (Z. Zhang et al. 2018; Zhao et al. 2021; Zhu et al. 2022). To have a more understanding of the subcellular localization of PvGATA28, a GFP fusion construct with the coding sequence of *PvGATA28* was prepared for this analysis. Using confocal laser microscopy, the GFP-PvGATA28 fusion was observed specifically within the nucleus of *Nicotiana benthamiana* leaf cells, as illustrated in Fig. 8. This observation aligns with our initial in-silico predictions, confirming the nuclear localization of PvGATA28.

This study also suggests potential functional differences among the subfamilies of *PvGATA* genes in different stress conditions. Identifying specific *PvGATA* genes that are up or down-regulated in response to stress may provide a foundation for breeding common bean plants with improved stress tolerance. Additionally, the study highlights the potential importance of the *GATA* gene family in regulating stress responses in common bean plants and provides insights into the molecular mechanisms underlying stress tolerance in plants. The identified *PvGATA* genes, specifically *PvGATA28*, holds immense promise in the development of common bean varieties with enhanced environmental stress tolerance. These genes could serve as one of the pivotal targets for breeding approaches aiming to enhance tolerance in common bean cultivars under challenging environmental conditions.

## Conclusion

In conclusion, this study provides insights into the functional identity of the *GATA* gene family in common bean plants. 31 GATA transcription factors were identified in *P. vulgaris*. This number was close to that found in *Arabidopsis* and rice. Gene duplication is also essential in the expansion of the *GATA* gene family in the common bean plant, and our phylogenetic analysis identified four subfamilies of *PvGATAs*. Members of subfamily II contained additional domains, such as CCT and tify domains, which have been observed to play a critical role in diverse biological functions in other plants. Our analysis of promoter *cis*-regulatory elements predicted 799 elements in all the *PvGATAs*. Five genes were selected for qRT-PCR expression analysis. Results indicated that *PvGATAs* may affect initial expression under abiotic stress. This study provides a basis for further functional studies on *PvGATAs* regulating abiotic stress tolerance and growth and development in common bean plants.

### Electronic supplementary material

Below is the link to the electronic supplementary material.

Supplementary Table S1: Primers used in qRT-PCR for expression analysis and overexpression of PvGATA28

Supplementary Table S2: Number of cis-regulatory elements on putative promoter region of PvGATA

Supplementary Table S3: Functioanl Gene ontology annotation analysis of PvGATA

Supplementary Table S4: Sub-cellular localization of PvGATA proteins, Diffrential expression of PvGATA transcripts from different tissues (Log2(FPKM)), and Diffrential expression of PvGATA transcripts under different stress conditions (Log2(FC))

Supplementary Table S5: One-to-one orthologous relationships between common bean and Arabidopsis and rice. And thier Phytozome transcript ID and corresponding gene names

## Data Availability

All data generated or analyzed during this study are included in this published article (and its Supporting Information files). The materials used in our study are available under an MTA from the corresponding author upon reasonable request.
